# Down-regulated FST expression is involved in the poor prognosis of triple-negative breast cancer

**DOI:** 10.1186/s12935-021-01977-x

**Published:** 2021-05-17

**Authors:** Sainan Liu, Bin Liu, Qian Zhao, Jikang Shi, Yulu Gu, Yanbo Guo, Yong Li, Yunkai Liu, Yi Cheng, Yichun Qiao, Yawen Liu

**Affiliations:** 1 Department of Epidemiology and Biostatistics, School of Public Health, Jilin University, Changchun, 130021 China; 2 Department of Breast Surgery, Second Affiliated Hospital of Jilin University, Changchun, 130021 China; 3 Institute of Translational Medicine, The First Hospital of Jilin University, Changchun, 130021 China

**Keywords:** Follistatin, Triple-negative breast cancer, Prognosis biomarker, Proliferation, Migration, Invasion

## Abstract

**Background:**

Triple-negative breast cancer (TNBC) is more commonly associated with young patients, featuring high histological grade, visceral metastasis, and distant recurrence. Follistatin (FST) is a secreted extracellular regulatory protein that antagonizes TGF-β superfamily such as activin and TGF-β related superfamily such as bone morphogenetic protein (BMP). The implication of FST in the proliferation, angiogenesis, and metastasis of solid tumors documents good or poor outcome of patients with BC. However, the role of FST in TNBC remains unclear.

**Methods:**

Data of 935 patients with breast cancer (BC) were extracted from TCGA. Case–control study, Kaplan–Meier, uni-multivariate COX, and ROC curve were utilized to investigate the relationship between FST expression and the clinical characteristics and prognosis of BC. Functional studies were used to analyze the effect of FST expression on proliferation, apoptosis, migration, and invasion of TNBC cell lines. Bioinformatic methods such as volcanoplot, GO annd KEGG enrichment, and protein–protein interactions (PPI) analyses were conducted to further confirm the different roles of FST in the apoptotic pathways among mesenchymal and mesenchymal stem-like cells of TNBC.

**Results:**

Data from TCGA showed that low FST expression correlated with poor prognosis (for univariate analysis, HR = 0.47, 95% CI: 0.27–0.82, *p* = 0.008; for multivariate analysis, HR = 0.40, 95% CI: 0.21–0.75, *p* = 0.004). Low FST expression provided high predicted value of poor prognosis in TNBC amongst BCs. FST knockdown promoted the proliferation, migration and invasion of BT549 and HS578T cell lines. FST inhibited the apoptosis of mesenchymal cells by targeting BMP7.

**Conclusions:**

Low FST expression is associated with poor prognosis of patients with TNBC. FST expressions exhibit the anisotropic roles of apoptosis between mesenchymal and mesenchymal stem-like cells but promote the proliferation, migration, invasion in both of two subtypes of TNBC in vitro. FST may be a subtype-heterogeneous biomarker for monitoring the progress of TNBC.

**Supplementary Information:**

The online version contains supplementary material available at 10.1186/s12935-021-01977-x.

## Background

BC, one of the most common cancers, is leading to a serious public health problem globally [1]. Based on the histology and molecular analysis, five main intrinsic or molecular subtypes have been identified: luminal A BC (estrogen-receptor [ER] and/or progesterone-receptor [PR] positive, human epidermal growth factor receptor [HER2] negative, and Ki-67 ≤ 14%), luminal B BC (ER and/or PR positive, and either HER2 positive or HER2 negative with PR < 20% and Ki-67 > 14%), triple-negative/basal-like BC (TNBC) (ER, PR and HER2 negative), HER-2-positive BC (ER and PR negative, and HER2 positive), and normal-like BC (similar to normal mammary gland) [2–5]. Luminal A and B BCs are also called hormone receptor (HR) positive BC [6]. Among these BCs, TNBC is usually found in young patients, featuring high histological grade, visceral metastasis, and distant recurrence. Currently, chemotherapy is the only treatment for TNBC. However, patients with TNBC usually have poor clinical outcomes with an overall response rates (ORRs) of 10–35%, and only 2–4 months of progression-free survival (PFS). Therefore, identifying actionable molecular targets is necessary for the pathogenesis and prognosis of TNBC [7].

Abnormalities in gene expression are involved in the progression of BC. Follistatin (FST) is a secreted extracellular regulatory protein that antagonizes TGF-β superfamily such as activin and TGF-β related superfamily such as bone morphogenetic protein (BMP) [8, 9]. FST has been reported to regulate hormone secretion, cell energy balance, tissue proliferation, differentiation, and metastasis to bone [10–12]. FST has been found to be implicated in the proliferation, angiogenesis, metastasis, and can be associated with good or poor outcome in solid tumors, including lung cancer [13], thymic epithelial tumors [14], and breast cancer [15]. FST overexpression induces apoptosis of MCF-7 cells [16] and suppresses metastasis in a mouse model of HER2-positive breast cancer [17]. However, the relationship between FST and TNBC remains unclear. In this paper, we investigated the involvement of FST in the pathogenesis and prognosis of TNBC.

## Methods

### Data and sources

Gene express data and clinical information of breast cancer were obtained from Gene expression-based Outcome for Breast cancer Online (GOBO) (http://co.bmc.lu.se/gobo/), The Cancer Genome Atlas (TCGA) (https://cancergenome.nih.gov/), Kaplan Meier-plotter (http://kmplot.com/analysis/) and Oncomine (https://www.oncomine.org/resource/login.html) online. The set of sequence-based mRNA expression data (RNA-seq data) and clinical information of breast cancer patients (n = 935) were downloaded from TCGA. A web-based tool, Metascape (http://metascape.org/), was employed to gain insights into the biological functions of differentially expressed genes between BT549 and HS578T cells.

### Cell culture

T47D, MCF7, BT549, and HS578T were procured from FuHeng Biology (FuHeng, China) and authenticated by STR (short tandem repeat) matching analysis. LCC2 was induced by MCF7 to form a stable cell line treated with low dose of tamoxifen for 6 months. MCF-7, LCC2, and HS578T cells were cultured in Dulbecco’s modified Eagle’s medium (DMEM, BioInd, Israel) supplemented with 10% fetal bovine serum (FBS, BioInd, Israel), and 1% penicillin/streptomycin (P/S, BioInd, Israel). T47D and BT549 cells were cultured in RPMI-1640 (BioInd, Israel) supplemented with 10% FBS, and 1% P/S. All these cells were incubated at 37 °C in a humidified atmosphere with 5% CO_2_.

### RNA interference

siRNAs targeting FST (si_FST) and negative control siRNA (si_Ctrl) were purchased from Ambion (Ambion, USA). BT549 and HS578T cells were transfected with si_FST and si_Ctrl at a final concentration of 10 μM/well and interferin transfection reagent (Polyplus) according to the manufacturer’s recommendations when the cells reached 40–60% confluency. After the cells were incubated for 24 h at 37 °C after transfection, the culture media were replaced with fresh DMEM or RPMI-1640 supplemented with 10% FBS, and 1% penicillin/streptomycin. The cells were harvested for quantitative real-time PCR and *in-vitro* experiments at 48 h after transfection, or Western blot at 72 h after transfection.

### RNA extraction and quantitative real-time PCR

Total RNA was extracted using EasyPure RNA Kit (TransGen, China). A total of 2 μg total RNA was reverse-transcribed into cDNA using FastKing gDNA Dispelling RT SuperMix (TIANGEN, China) according to the manufacturer’s instructions. Real-time PCR was performed on Bio-Rad CFX96 Real-Time System using SuperReal PreMix Plus kit (TIANGEN, China) according to the conditions specified by the manufacture. Quantitation of 36B4 was used as an internal control. The relative expression level was calculated using comparative Ct. Primer sequences are listed in Additional file 2: Table S2.

### Protein extraction and Western Blot

Total protein was isolated using extraction kit (BestBio, China) and measured using BCA Protein Assay Kit (Beyotime, China). Samples were separated by 10% SDS PAGE gels and transferred to a polyvinylidene fluoride membrane (PALL, USA). The membrane was blotted with rabbit lgG antibody against FST (1:500 dilution) (Sigma-Aldrich, USA) and probed with mouse anti-rabbit IgG antibody (1:10,000 dilution) (GE Healthcare, UK). The protein levels were normalized by β-actin probing with β-actin antibody (1:10,000 dilution) (Sigma-Aldrich, USA) and sheep anti-mouse IgG antibody (1:10,000 dilution) (GE Healthcare, UK). Immunosignals were imaged using a Tanon 5,200 Multi Automatic Chemiluminescence / Fluorescence Image Analysis System (China).

### Cell proliferation assay

Cell proliferation was measured using MTS (SAINT-BIO, China) according to the manufacturer’s instructions. Briefly, BT549 or HS578T transfected with FST siRNAs were seeded into 96-well plates (1,000 cells/well). A total of 10 μl of MTS reagent was added to the test well. After incubating for 2 h, the absorbance was measured at 490 nm using a multilabel plate reader (Thermo, USA).

### Cell apoptosis assay

Cell apoptosis was detected by FITC Annexin V Apoptosis Detection Kit I (BD Biosciences, USA) according to the manufacturer’s instructions. Cells were harvested and washed two times using phosphate-buffered saline and resuspended in 1 × Binding Buffer at a final concentration of 1–5 × 10^6^ cells/mL. 100 μl resuspended cells were stained with PI and FITC for 15 min at room temperature in darkness. Finally, the cells were analyzed using a FACS Calibur (BD Biosciences, USA) after adding 400 μl 1 × Binding Buffer.

### Cell migration assay

Wound-healing assay was performed to assess cell migration. When cells reached 90% confluence, cells were further exposed for 2 h to a medium supplemented with 10 µg/ml Mitomycin-C (Roche, China). After the wound was formed, the wound was flushed with PBS to remove debris. Then the PBS was replaced with a serum-free medium for continuous culture [18, 19]. The recovery of wound was observed and captured under a microscope at 6, 12, and 24 h after scratch. Degree of migration for each time point per experiment was determined by calculating the average pixel area of the three fields in duplicate [20].

### Cell invasion assay

A total of 10^5^ cells were seeded in the upper chamber of 24-well Growth Factor Reduced Corning Matrigel Invasion chamber (Corning, USA) with serum-free media. Media containing 10% FBS and 1% P/S were added to the lower chamber. After 24 h, the cells in the upper chamber were scraped by a cotton swab wetted by PBS. Cells invading to the lower chamber were fixed, stained, and counted under a microscope.

### Statistical analysis

Microsoft EXCEL and IBM SPSS 24.0 (SPSS Inc., Chicago, IL, USA) were used to perform χ^2^ [1] test and fisher exact probability test to compare the correlation of FST expression with clinicopathologic characteristics in breast cancer patients. Univariate and multiple binary logistic regression Overall and relapse-free survival curves were calculated according to the Kaplan–Meier method, and comparison was performed using the log-rank test. Univariate and multiple COX analysis were used to reveal the association between FST expression and survival. The variables with *p* < 0.01 in univariate analysis were considered as covariables adjusted in multiple analysis. Two-way ANOVA and two-tailed Student’s t-test were used in cell studies. ROC curves and expression cutoff values were performed using R (Version 3.5.2) in R studio. *p* values < 0.05 were taken to indicate statistical significance.

## Results

### FST expressions are significantly associated with prognosis of patients with breast cancer

**Fig. 1:**
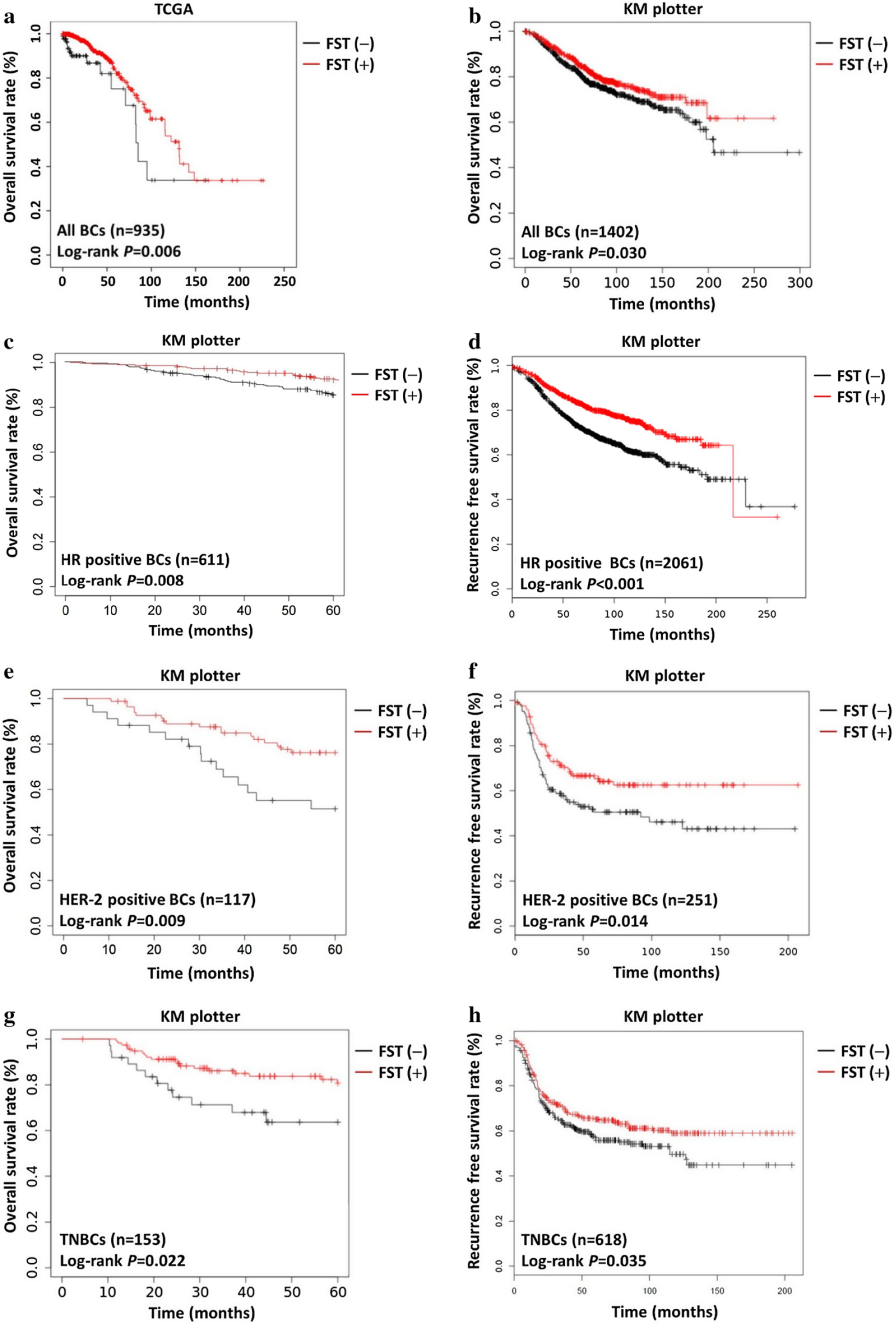
Survival analysis of all and molecular subtypes of patients with BCs based on FST expression. **a**, **b** Kaplan–Meier OS analysis of FST expression for all patients in TCGA. **a** and Kaplan–Meier plotter (**b**). **c**–**h** Kaplan–Meier OS and RFS analysis of FST expression for patients with HR positive BCs (include luminal A and luminal B BCs) **c**, **d** HER-2 positive BCs **e**, **f** and TNBCs **g**, **h** in Kaplan–Meier plotter

To identify the relationship between FST expressions and clinical characteristics (pathological stage, tumor size, lymph node, distant metastasis, ER status, PR status, HER-2 status, molecular subtype, histological type and survival status) in breast carcinogenesis, the mRNA-seq data of 935 BC patients were recruited from the TCGA database. According to current classified guideline for BC molecular subtype, it is imprecise to distinguish luminal A and/or luminal B subtype without indicator of ki-67 in TCGA. HR positive BC was used to integrated luminal A and luminal B BC in Tables 1, 2, 3, and Additional file 1: Table S1. FST expressions were found to be positively associated with the molecular subtype, histological type and survival status of BCs (Table 1). Non-conditional logistic analysis further revealed that low FST expressions correlated with risks of HER-2 positive BC (OR = 0.38, 95% CI: 0.16–0.88), TNBC (OR = 0.71, 95% CI: 0.35–1.43), Mucinous BC (OR = 0.17, 95% CI: 0.06–0.48) tumors, and dead status (OR = 0.50, 95% CI: 0.27–0.93) (Additional file 1: Table S1). We next analyzed data from TCGA and Kaplan Meier-plotter database, finding that patients with high FST expressions had better OS than patients with low FST expressions (Fig. 1a, b, log-rank *p* < 0.05). Moreover, trends of Kaplan Meier-plotter documented much more benefit in patients with HER-2 positive BC and TNBC than in those with HR positive BC.

**Table 1:** Relationship between FST expression and clinicopathologic characteristics in BCs from TCGA. [N = 935]

Characteristics	No. of patients	Number of positive FST (%)	*p*-value
Age			0.066
≤ 45	153	144 (94.1)	
> 45	782	698 (89.3)	
Ethnicity^#^			0.521
Hispanic/Latino	32	28 (87.5)	
Not Hispanic/Latino	744	676 (90.9)	
Pathological stage^#^			0.415
I/II	689	618 (89.7)	
III/IV	236	216 (91.5)	
Tumor size			0.843
T1/T2	776	699 (90.1)	
T3/T4	149	135 (90.6)	
Lymph node^#^			0.143
Negative	427	379 (88.8)	
Positive	490	449 (91.6)	
Distant metastasis^#^			0.845
Negative	771	696 (90.3)	
Positive	18	16 (88.9)	
ER status^#^			0.659
Negative	264	240 (90.9)	
Positive	667	600 (90.0)	
PR status^#^			0.260
Negative	161	149 (92.5)	
Positive	772	692 (89.6)	
HER2 status^#^			1.000
Negative	531	472 (88.9)	
Positive	162	144 (88.9)	
Molecular subtype			0.029*
HR positive BC	808	734 (90.8)	
HER–2 positive BC	36	28 (77.8)	
TNBC	91	80 (87.9)	
Histological subtype^#^			0.003*
Infiltrating ductal BC	655	590 (90.1)	
Infiltrating lobular BC	192	180 (93.8)	
Mixed BC	24	23 (95.8)	
Medullary BC	3	3 (100.0)	
Metaplastic BC	5	4 (80.0)	
Mucinous BC	16	10 (62.5)	
Others	39	31 (79.5)	
Survival status			0.011*
Alive	851	773 (90.8)	
Dead	84	69 (82.1)	

^#^The composition ratio is less than 100%; *p < 0.05, statistical significance

**Table 2:** Univariate and multiple COX analyses of overall survival in BC patients

Characteristics	N	Univariate analysis		Multiple analysis	
		HR (95% CI)	*p*-value	HR (95% CI)	*p*-value
Age					
≤ 45	153	Ref		Ref	
> 45	782	1.46 (0.81–2.65)	0.208	1.68 (0.88–3.20)	0.117
Ethnicity^#^					
Hispanic/Latino	32	Ref		Ref	
Not Hispanic/Latino	744	21.83 (0.24–9.29)	0.178	NA	0.965
Pathological stage^#^					
I/II	707	Ref		Ref	
III/IV	239	2.28 (1.45–3.61)	0.000*	1.51 (0.81–2.79)	0.195
Tumor size					
T1/T2	797	Ref		Ref	
T3/T4	157	1.39 (0.83–2.33)	0.207	0.89 (0.44–1.80)	0.753
Lymph node^#^					
Negative	444	Ref		Ref	
Positive	494	2.04 (1.26–3.30)	0.004*	1.77 (0.97–3.20)	0.061
Distant metastasis^#^					
Negative	788	Ref		Ref	
Positive	18	4.35 (2.16–8.75)	0.000*	2.54 (1.11–5.83)	0.028*
ER status^#^					
Negative	264	Ref		Ref	
Positive	667	1.02 (0.63–1.67)	0.925	0.99 (0.58–1.71)	0.981
PR status^#^					
Negative	161	Ref		Ref	
Positive	772	2.88 (1.17–7.12)	0.022*	2.78 (1.11–6.98)	0.029*
HER2 status^#^					
Negative	531	Ref		Ref	
Positive	162	0.96 (0.54–1.71)	0.891	0.66 (0.34–1.30)	0.230
Molecular subtype					
HR positive BC	808	Ref		Ref	
HER-2 positive BC	36	2.68 (1.07–6.76)	0.036*	2.24 (0.80–6.30)	0.127
TNBC	91	1.67 (0.90–3.11)	0.104	1.95 (1.01–3.77)	0.047*
Histological subtype^#^					
Infiltrating ductal BC	655	Ref		Ref	
Infiltrating lobular BC	192	0.29 (0.12–0.66)	0.003*	0.42 (0.18–0.99)	0.049*
Mixed BC	24	0.56 (0.14–2.31)	0.426	0.21 (0.03–1.57)	0.129
Medullary BC	3	1.03 (0.25–4.32)	0.963	1.68 (0.39–7.14)	0.484
Metaplastic BC	5	NA	0.977	NA	0.974
Mucinous BC	16	2.18 (0.30–15.85)	0.441	3.86 (0.52–28.64)	0.187
Others	39	1.07 (0.49–2.35)	0.872	0.99 (0.37–2.62)	0.983
FST expression					
Low	77	Ref		Ref	
High	858	0.47 (0.27–0.82)	0.008*	0.40 (0.21–0.75)	0.004*

^#^The composition ratio is less than 100%; *p < 0.05, statistical significance

*HR* hazard ratio, *CI* confidence interval, HR positive BC, hormone receptor positive breast cancer; HER-2 positive BC, human epidermalgrowth factor receptor-2 positive breast cancer; *TNBC* triple-negative breast cancer

**Table 3:** Stratification and uni–multivariate analysis based on FST expression for survival of BCs

Characteristics	No. of cases	HR of OS (95% CI)	*p*-value	Adjusted	Adjusted
				HR of OS^a^ (95% CI)	*p*-value
Pathological stage^#^					
I/II	689	0.45 (0.22–0.93)	0.031*	0.32 (0.15–0.70)	0.004*
III/IV	236	0.37 (0.14–0.97)	0.043*	0.49 (0.17–1.46)	0.203
Lymph node^#^					
Negative	427	0.37 (0.15–0.89)	0.026*	0.23 (0.09–0.62)	0.003*
Positive	490	0.35 (0.15–0.84)	0.018*	0.46 (0.18–1.19)	0.109
Distant metastasis^#^					
Negative	771	0.41 (0.22–0.75)	0.004*	0.35 (0.18–0.68)	0.002*
Positive	18	0.67 (0.08–5.63)	0.713	0.83 (0.10–7.20)	0.867
PR status^#^					
Negative	161	NA	0.809	NA	0.996
Positive	772	0.49 (0.28–0.86)	0.013*	0.42 (0.23–0.78)	0.006*
Molecular subtype					
HR positive	808	0.46 (0.25–0.85)	0.012*	0.39 (0.21–0.75)	0.005*
HER–2 positive	36	0.19 (0.03–1.42)	0.105	0.26 (0.04–1.97)	0.194
TNBC	91	3.85 (0.83–17.93)	0.086	6.39 (0.78–52.34)	0.084
Histological subtype^#^					
Infiltrating ductal BC	655	0.40 (0.21–0.78)	0.007*	0.43 (0.21–0.90)	0.025*
Infiltrating lobular BC	192	0.29 (0.04–1.89)	0.194	0.08 (0.01–0.57)	0.012*
Mixed BC	24	NA	NA	NA	NA
Medullary BC	3	NA	NA	NA	NA
Metaplastic BC	5	NA	NA	NA	NA
Mucinous BC	16	NA	NA	NA	NA
Others	39	0.72 (0.13–3.98)	0.703	0.77 (0.14–4.33)	0.770

^a^Ajusted HR, adjusted by age and ethnicity in COX analysis; ^#^The composition ratio is less than 100%; *p < 0.05, statistical significance

**Fig. 2:**
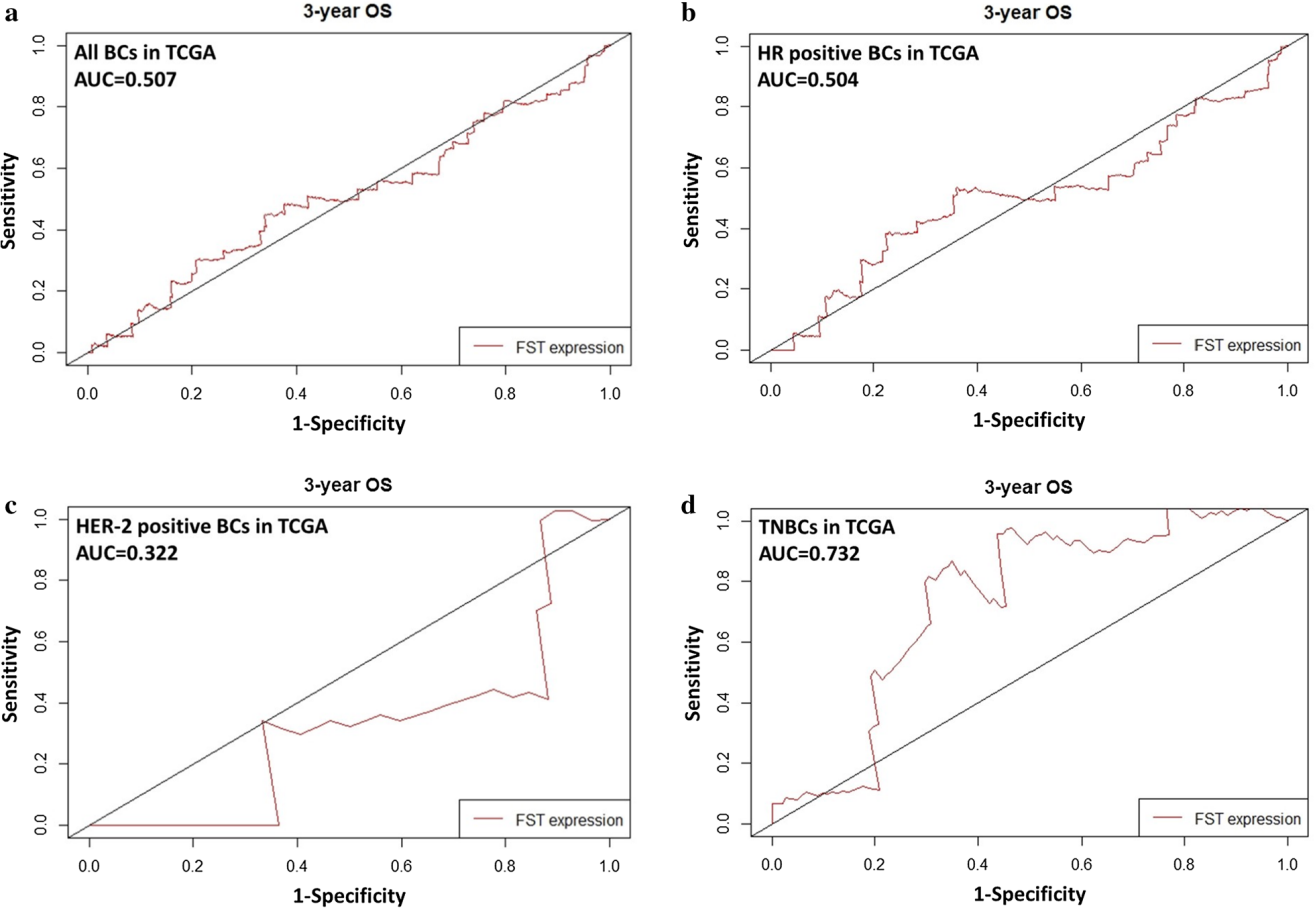
Prognostic value of FST expressions in patients with BCs. **a** Time-dependent ROC analysis of 3-year OS for pantients with BCs (AUC = 0.507), **b** HR positive BCs (AUC = 0.504), **c** HER-2 positive BCs (AUC = 0.322) and **d** TNBCs (AUC = 0.732) in TCGA

Low FST expressions significantly correlated with reduced overall survivals (Fig. 1c, e, g, log-rank* p* < 0.05) and relapse-free survivals (Fig. 1d, f, h, log-rank* p* < 0.05) in patients with HR-positive BC, HER2-positive BC, or TNBC. ROC curves showed that different predicting values for BC patients (Fig. 2a–d, for all BCs, AUC = 0.507; for HR-positive BCs, AUC = 0.504; for HER-2 positive BCs, AUC = 0.322; for TNBCs, AUC = 0.732). As shown in Table 2, univariate COX analysis exhibited that worse OS was associated with pathological stage III-IV than I-II (HR = 2.28, 95% CI: 1.45–3.61), lymph node involvement than non-involvement (HR = 2.04, 95% CI: 1.26–3.30), distant metastasis positive than negative (HR = 4.35, 95% CI: 2.16–8.75), PR positive than negative (HR = 2.88, 95% CI: 1.17–7.12), HER-2 positive BC than HR positive BC (HR = 2.68, 95% CI: 1.07–6.76), infiltrating ductal BC than infiltrating lobular BC (HR = 0.29, 95% CI: 0.12–0.66), and low than high FST expression (HR = 0.47, 95% CI: 0.27–0.82). Furthermore, multivariate COX analysis documented that high FST expression (HR = 0.40, 95% CI: 0.21–0.75) and infiltrating lobular BC (HR = 0.42, 95% CI: 0.18–0.99) were favorable independent prognostic predictors for OS. However, distant metastasis (HR = 2.54, 95% CI: 1.11–5.83), PR positive (HR = 2.78, 95% CI: 1.11–6.98), and TNBC subtype (HR = 1.95, 95% CI: 1.01–3.77) were poor independent prognostic predictors for OS. Moreover, stratified Kaplan–Meier analysis showed that increased expression of FST was good predictors for survival in pathological stage I–II (Additional file 3: Fig. S1a, log-rank* p* < 0.05), distant metastasis free (Additional file 3: Fig. S1b, log-rank* p* < 0.01), lymph node free (Additional file 3: Fig. S1c, d, log-rank* p* < 0.05), and PR-positive subgroups of patients with BC (Additional file 3: Fig. S1e, f, log-rank* p* < 0.05). Stratified uni–multivariate analysis adjusted by ethnicity and age for survival of BCs further validated the results from Kaplan–Meier analysis in Table 3. Patients with BC of pathological stage I–II (adjusted HR = 0.32, 95% CI: 0.15–0.70), lymph node free (adjusted HR = 0.23, 95% CI: 0.09–0.62), distant metastasis free (adjusted HR = 0.35, 95% CI: 0.18–0.68), PR positive (adjusted HR = 0.42, 95% CI: 0.23–0.78), molecular subtype of HR positive BC (adjusted HR = 0.39, 95% CI: 0.21–0.75), and histological type of infiltrating ductal (adjusted HR = 0.43, 95% CI: 0.21–0.90) or infiltrating lobular BC (adjusted HR = 0.08, 95% CI: 0.01–0.57) exhibited the consistent trend that high expression of FST was good predictors for survival. These results documented the association of FST expressions with the prognosis of breast cancer patients.

### Up-regulation of FST expression inclines to reflect low malignancy of BC

**Fig. 3:**
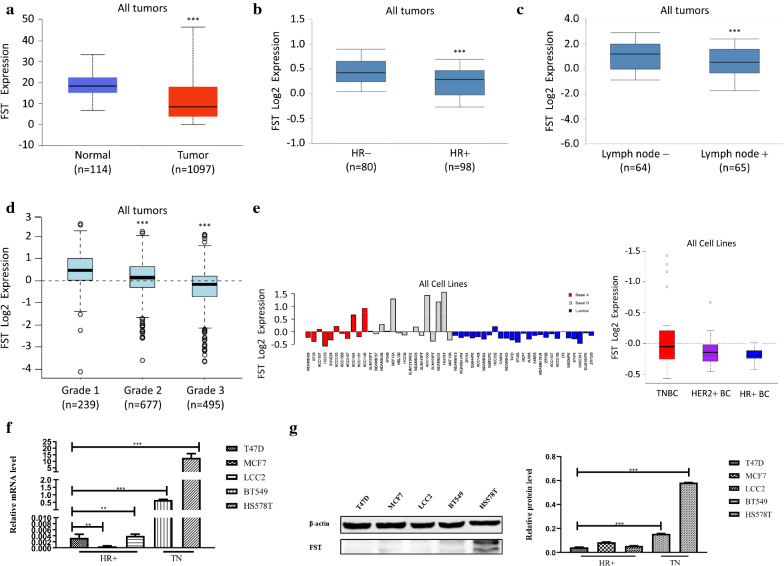
FST expressions in normal breast tissues and BC tumors with different clinical characteristics and in different BC cell lines. **a** FST mRNA expression in tumors is lower than that in normal breast tissues. **b**–**d** FST mRNA expression is higher in HR negative **b**, lymph node involvement **c** and low histological grade tumors **d** than HR positive, lymph node free and high histological grade ones. **e** FST mRNA expression in TNBC cell lines is the highest, followed by HER-2 positive and HR positive breast cancer cell lines. **f** FST mRNA expression and **g** FST protein expression in TNBC cell lines (BT549, HS578T) was significantly higher than cell lines of other molecular types (T47D, MCF7, LCC2). *p < 0.05, **p < 0.01, ***p < 0.001

We next analyzed FST expressions in BC tumors and cell lines using RNA–seq data from the TCGA, Oncomine, and GOBO dataset. We found that FST mRNA expressions in breast cancer tissues were significantly low compared with those in normal breast tissues (Fig. 3a,  *p*< 0.001), suggesting that low FST expression may be a risk factor for BC. Furthermore, FST mRNA expressions were higher in patients with HR negative, lymph node free and low histological grade tumors than those in their respective counterparts significantly (Fig. 3b–d,  *p*< 0.001). In addition, the level of FST mRNA in TNBC cell lines was the highest among all BC cell lines (Fig. 3e,* p* > 0.05). RT–qPCR and Western blot were further performed to confirm our analysis using five different BC cell lines (HR positive cell lines [T47D and MCF7], tamoxifen resistance cell line [LCC2], and TNBC cell lines [BT549 and HS578T]). The results showed that FST mRNA and protein were highly expressed in BT549 and HS578T cells compared with T47D, MCF7, and LCC2 (Fig. 3f, g,* p* < 0.001).

### Down-regulation of FST results in the acceleration of proliferation, migration, and invasion of TNBC cells

**Fig. 4:**
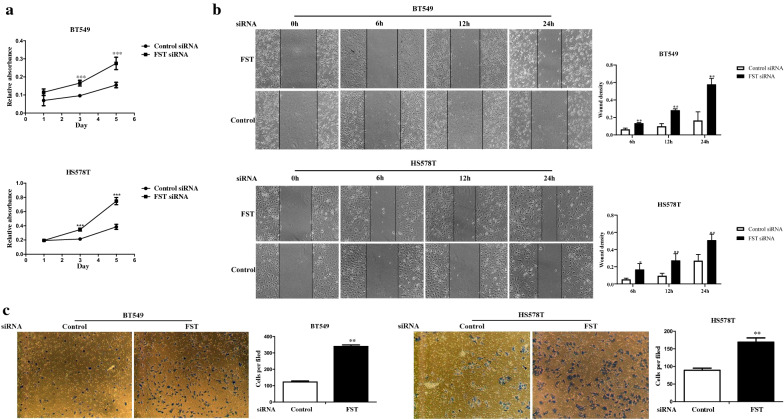
FST inhibited TNBC cell lines proliferation, migration and invasion, and promoted BT549 cell apoptosis. **a** Proliferation assays were assessed in BT549 and HS578T cells transfected with FST siRNA or control siRNA. **b** Wound healing assays for BT549-Control/FST and HS578T-Control/FST cells. **c** Matrigel invasion assays were assessed in BT549 and HS578T cells transfected with FST siRNA or control siRNA. *p < 0.05, **p < 0.01, ***p < 0.001

FST knockdown in BT549 and HS578T cells was performed to investigate the biological function of FST in TNBC (Additional file 4: Fig. S2a, b). Decreased FST expression significantly increased cell proliferation of TNBC cells (Fig. 4a). The wound–healing assay showed that wound densities were significantly increased in FST knockdown cells compared with those in control cells (Fig. 4b). Furthermore, the Matrigel invasion assay showed that FST knockdown significantly increased the invasive capacities of TNBC cells (Fig. 4c). We found that FST knockdown inhibited the apoptosis in BT549 cells but promoted the apoptosis in HS578T cells (Additional file 4: Fig. S2c). To validate the different signal pathways enriched in mesenchymal and mesenchymal stem-like breast cancer subtypes, we performed bioinformatic analysis on the basis of GEO dataset. The results showed that the gene expression levels of BT549 on apoptosis-related pathway were significantly higher than those of HS578T cell line (Additional file 4 Fig. S2d-f), and the expression level of BMP7 mRNA in BT549 cells was nearly seven times greater than that of HS578T cells (Additional file 4: Fig. S2g). On the contrary, the expression level of FST mRNA in BT549 cells was nearly 18 times less than that of HS578T cells (Fig. 3f). Moreover, the expression levels of BMP7 in both BT549 and HS578T cells were increased after FST knockdown suggesting that high expression of BMP7 in BT549 cell may be influenced by FST (Additional file 4: Fig. S2h). Taken together, these results corroborated that BMP7 is differentially implicated in FST regulation between BT549 and HS578T cells, and down-regulation of FST is involved in the acceleration of proliferation, migration, and invasion of TNBC cells.

## Discussion

In this paper, we identified that down-regulation of FST confers poor prognosis in patients with TNBC and down-regulation of FST expression inclines to reflect tumor growth of TNBC. This is the first study revealing anisotropic roles of FST expression in TNBC.

Associations of FST expression with BC malignancy have been widely investigated without discriminating BC molecular subtypes. Zabkiewicz C et al. found that FST expression correlates with pathological and prognostic parameters in a cohort of patients with BC from United Kingdom [10]. Wallner C et al. found that increased expression of FST is associated with a higher overall survival rate of patients with BC from Germany [21]. Couto HL revealed that FST expression in invasive BC is not related to the disease severity and the risk of recurrence in patients from Brazil [22]. Notably, Mock K found that high FST expression promotes bone metastasis using the GEO dataset [23]. In contrast to the studies mentioned above, we focused on TNBC and identified that high FST expression confers high prognostic value for patients with TNBC. Therefore, detail characterization of molecular subtypes of BC is needed for investigating associations between FST expression and BC prognosis.

FST is expressed in the normal mammary gland and proliferative breast disease [22]. For ER-positive BC, activin, a potent native FST antagonist, promotes the EMT (Epithelial mesenchymal transformation), and enhance the invasiveness of MCF–7. In contrast, the function of activin has been found to be abrogated to a greater degree by the addition of FST treatment in ER negative cells. For these reasons, differential FST roles are necessary for discerning the heterogeneous natures of breast cancer and its molecular subtypes.

In addition, FST over-expression prevents the EMT and invasion of breast epithelial cells by binding directly to TNF–β [21]. Knocking-down FST reduces the viability of the MCF7 cells and disrupts their migrant and proliferative potential [21]. For HER-2 positive BC, Darcie D et al. found that FST overexpression reduces metastasis of HER-2 positive BC in mice [17]. For TNBC, Ohta N et al. found that FST over-expression suppresses the growth of MDA-MB-231 cells [24], further providing evidence for our finding that down-regulated FST expression is associated with poor prognosis in TNBC.

TNBC is a highly heterogeneous disease. Recently, transcriptome-based subtypes of TNBCs such as: basal like-1, basal like-2, immunomodulatory, mesenchymal, mesenchymal stem-like, luminal androgen receptor, and unclassified have been identified [25]. We found that the expression of BMP7 in BT549 was nearly 7 times as much as that in HS578T. Moreover, BMP7, a key member of negative regulator to mesenchymal cell apoptotic process as well as a member of TGF-β related superfamily, is inhibited by FST [26]. For these reasons, BMP7 may be mainly involved in FST regulation in BT549 cells compared with that in HS578T cells. In addition, glucose, glutamine, lactate, pyruvate, and free FAs are essential for tumor growth [27]. Metabolic phenotypes of TNBC are different from those of luminal or HER2-enriched BC: cellular metabolism among subtypes of TNBC differs dramatically [28]. Furthermore, TNBCs can be classified into three metabolic-pathway-based subtypes with distinct metabolic features (the lipogenic subtype with upregulated lipid metabolism, the glycolytic subtype with upregulated carbohydrate and nucleotide metabolism, and the mixed subtype with partial pathway dysregulation) [29]. Both glycolysis and FA synthesis are essential for cancer cell proliferation. However, the glycolytic subtype of TNBC is associated with worse outcomes [29]. Therefore, glycolysis may function as a more important role in the pathogenesis of TNBC than other subtypes of BC. Notably, we found that down–regulated FST promoted proliferation, migration, and invasion of both HS578T and BT549 cells and inhibited apoptosis of BT549. The down–regulation of FST expression increased the apoptosis rate of HS578T cells by about 15%; however, the proliferation rate of HS578T cells doubled when compared to the control. Compared with RPMI-1640 medium where BT549 cultured, DMEM medium provided high glucose concentration for HS578T which may differentiate its growth status from other cell lines cultured in medium with low concentration of glucose. Thus, TNBC cell lines should be further investigated and classified using the combination of transcriptome-based subtypes and metabolic-pathway-based subtypes.

There are some limitations in this paper: Firstly, limited subtypes of TNBC cell lines were included in this study. Secondly, because TCGA only provided limited patients with complete clinical data, we should enroll more patients with complete clinical data in the future.

## Conclusion

In summary, FST expressions exhibit the anisotropic roles of apoptosis between mesenchymal and mesenchymal stem-like cells but promote the proliferation, migration, invasion in both of two subtypes of TNBC in vitro. FST expression confers high prognostic value for the survival of patients with TNBC. FST may be a subtype-heterogeneous biomarker for monitoring the progress of TNBC.

## Supplementary Information

**Additional file 1: Table S1.** Nonconditional logistic analysis for FST and some clinicopathologic characteristics.

**Additional file 2: Table S2.** Primer sequences of 36B4 and FST.

**Additional file 3: Figure S1.** Kaplan–Meier OS analysis of FST expression for patients with pathological stage I-II, distant metastasis free, lymph node free and PR positive BCs in TCGA and KM plotter.

**Additional file 4: Figure S2.** FST expression in siRNA targeted FST model and the role of FST in apoptotic pathways between BT549 and HS578T cells.

## Data Availability

The datasets used and/or analyzed during the current study are available from the corresponding author on reasonable request.

## Abbreviations

BC: Breast cancer
HR positive BC: Hormone receptor positive breast cancer
HER-2 positive BC: Human epidermal growth factor receptor-2 positive breast cancer
TNBC: Triple-negative breast cancer
ORRs: Overall response rates
PFS: Progression-free survival
FST: Follistatin
OS: Overall survival
RFS: Relapse-free survival
OR: Odds ratio
HR: Hazard ratio
CI: Confidence interval
