# Supplementary material for: Down-regulated FST expression is involved in the poor prognosis of triple-negative breast cancer
Source: Cancer Cell Int. 2021 May 17;21:267. doi: 10.1186/s12935-021-01977-x (PMC8130405; doi:10.1186/s12935-021-01977-x)
Supplement: Supplementary file 1 — Additional file 1: Table S1. Nonconditional logistic analysis for FST and some clinicopathologic characteristics. [file 12935_2021_1977_MOESM1_ESM.docx]

**Table S1** Nonconditional logistic analysis for FST and some clinicopathologic characteristics.

| Characteristics | *p*–value | OR(95%CI) | Adjusted  *p*–value | Adjusted OR(95%CI) |
| --- | --- | --- | --- | --- |
| **Age** |  |  |  |  |
| Ⅰ/Ⅱ |  | Ref |  | Ref |
| Ⅲ/Ⅳ | 0.071 | 0.52(0.26-1.06) | 0.068 | 0.51(0.25-1.05) |
| **Molecular subtype** |  |  |  |  |
| HR positive BC |  | Ref |  | Ref |
| HER-2 positive BC | 0.013^*^ | 0.35(0.16-0.80) | 0.023^*^ | 0.38(0.16-0.88) |
| TNBC | 0.367 | 0.73(0.37-1.44) | 0.337 | 0.71(0.35-1.43) |
| **Histological type^#^** |  |  |  |  |
| Infiltrating ductal BC |  | Ref |  | Ref |
| Infiltrating lobular BC | 0.123 | 1.65(0.87-3.13) | 0.228 | 1.50(0.78-2.88) |
| Mixed BC | 0.367 | 2.53(0.34-19.07) | 0.428 | 2.27(0.30-17.24) |
| Medullary BC | 0.999 | NA | 0.999 | NA |
| Metaplastic BC | 0.467 | 0.44(0.05-4.00) | 0.592 | 0.54(0.06-5.13) |
| Mucinous BC | 0.001^*^ | 0.18(0.07-0.52) | 0.001^*^ | 0.17(0.06-0.48) |
| Others | 0.041^*^ | 0.43(0.19-0.97) | 0.054 | 0.44(0.19-1.01) |
| **Survival status** |  |  |  |  |
| Alive |  | Ref |  | Ref |
| Dead | 0.013^*^ | 0.46(0.25-0.85) | 0.029^*^ | 0.50(0.27-0.93) |

**Notes:** ^a^ Ajusted OR, adjusted by parameters with *p* < 0.10 in Table 1; **^#^** The composition ratio is less than 100%;^*^ *p* < 0.05, statistical significance.
