# Supplementary material for: Down-regulated FST expression is involved in the poor prognosis of triple-negative breast cancer
Source: Cancer Cell Int. 2021 May 17;21:267. doi: 10.1186/s12935-021-01977-x (PMC8130405; doi:10.1186/s12935-021-01977-x)
Supplement: Supplementary file 2 — Additional file 2: Table S2. Primer sequences of 36B4 and FST. [file 12935_2021_1977_MOESM2_ESM.docx]

**Table 3** Stratification and uni–multivariate analysis based on FST expression for survival of BCs

| Characteristics | No. of cases | HR of OS(95%CI) | *p*–value | Adjusted | Adjusted |
| --- | --- | --- | --- | --- | --- |
|  |  |  |  | HR of OS^a^(95%CI) | *p*–value |
| **Pathological stage^#^** |  |  |  |  |  |
| Ⅰ/Ⅱ | 689 | 0.45(0.22-0.93) | 0.031^*^ | 0.32(0.15-0.70) | 0.004^**^ |
| Ⅲ/Ⅳ | 236 | 0.37(0.14-0.97) | 0.043^*^ | 0.49(0.17-1.46) | 0.203 |
| **Lymph node^#^** |  |  |  |  |  |
| Negative | 427 | 0.37(0.15-0.89) | 0.026^*^ | 0.23(0.09-0.62) | 0.003^*^ |
| Positive | 490 | 0.35(0.15-0.84) | 0.018^*^ | 0.46(0.18-1.19) | 0.109 |
| **Distant metastasis^#^** |  |  |  |  |  |
| Negative | 771 | 0.41(0.22-0.75) | 0.004^*^ | 0.35(0.18-0.68) | 0.002^*^ |
| Positive | 18 | 0.67(0.08-5.63) | 0.713 | 0.83(0.10-7.20) | 0.867 |
| **PR status^#^** |  |  |  |  |  |
| Negative | 161 | NA | 0.809 | NA | 0.996 |
| Positive | 772 | 0.49(0.28-0.86) | 0.013^*^ | 0.42(0.23-0.78) | 0.006^*^ |
| **Molecular subtype** |  |  |  |  |  |
| HR positive | 808 | 0.46(0.25-0.85) | 0.012^*^ | 0.39(0.21-0.75) | 0.005^*^ |
| HER–2 positive | 36 | 0.19(0.03-1.42) | 0.105 | 0.26(0.04-1.97) | 0.194 |
| TNBC | 91 | 3.85(0.83-17.93) | 0.086 | 6.39(0.78-52.34) | 0.084 |
| **Histological subtype^#^** |  |  |  |  |  |
| Infiltrating ductal BC | 655 | 0.40(0.21-0.78) | 0.007^*^ | 0.43(0.21-0.90) | 0.025^*^ |
| Infiltrating lobular BC | 192 | 0.29(0.04-1.89) | 0.194 | 0.08(0.01-0.57) | 0.012^*^ |
| Mixed BC | 24 | NA | NA | NA | NA |
| Medullary BC | 3 | NA | NA | NA | NA |
| Metaplastic BC | 5 | NA | NA | NA | NA |
| Mucinous BC | 16 | NA | NA | NA | NA |
| Others | 39 | 0.72(0.13-3.98) | 0.703 | 0.77(0.14-4.33) | 0.770 |

**Notes:** a Ajusted HR, adjusted by age and ethnicity in COX analysis; # The composition ratio is less than 100%; **p* < 0.05, statistical significance.
