# Supplementary figures and images for: Down-regulated FST expression is involved in the poor prognosis of triple-negative breast cancer
Source: Cancer Cell Int. 2021 May 17;21:267. doi: 10.1186/s12935-021-01977-x (PMC8130405; doi:10.1186/s12935-021-01977-x)

a

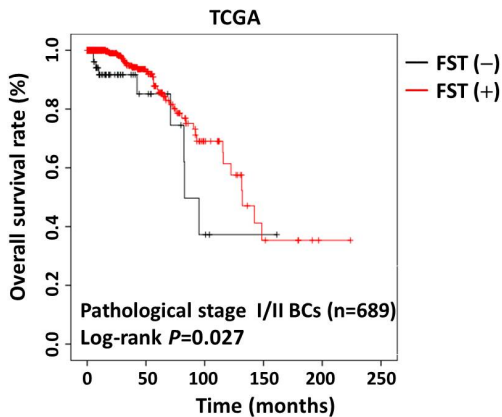

b

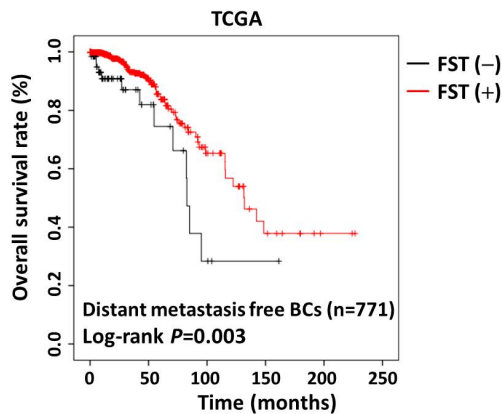

c

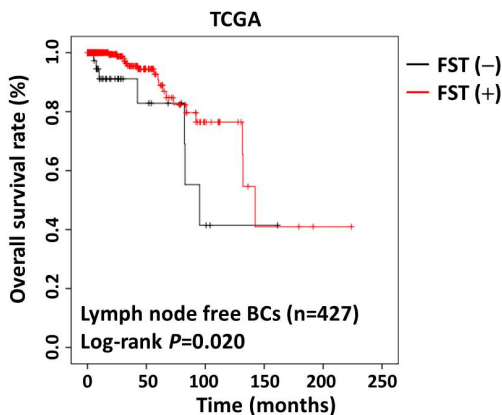

d

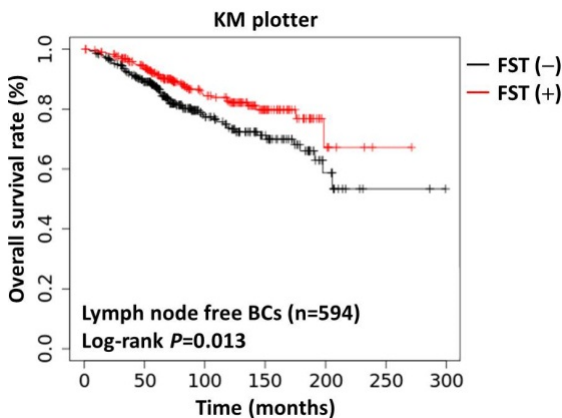

e

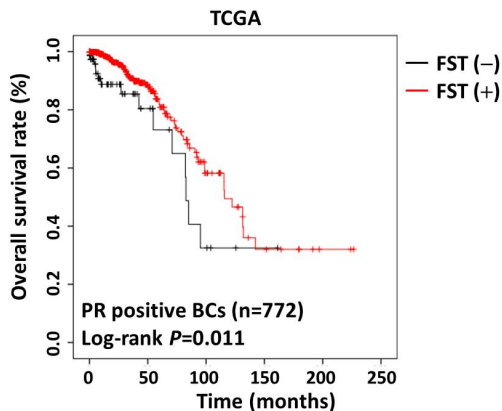

f

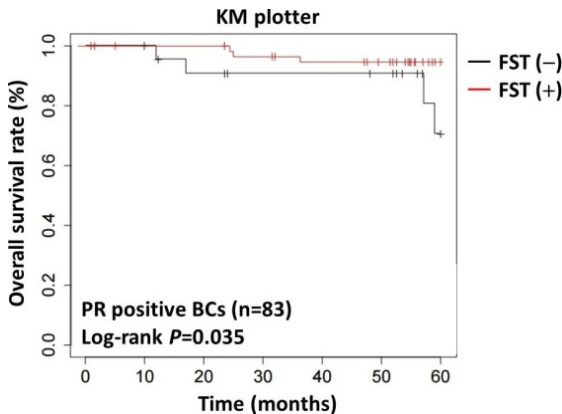

Supplement: Supplementary file 3 — Additional file 3: Figure S1. Kaplan–Meier OS analysis of FST expression for patients with pathological stage I-II, distant metastasis free, lymph node free and PR positive BCs in TCGA and KM plotter. [file 12935_2021_1977_MOESM3_ESM.pdf]

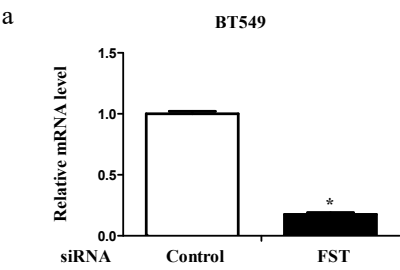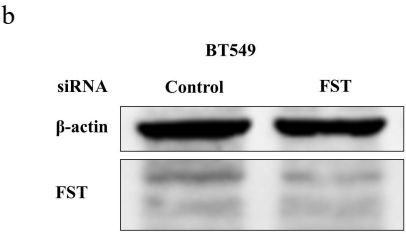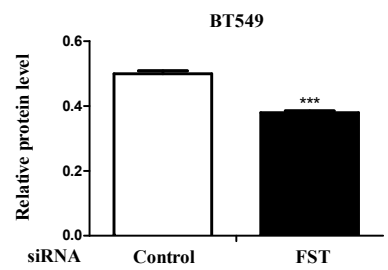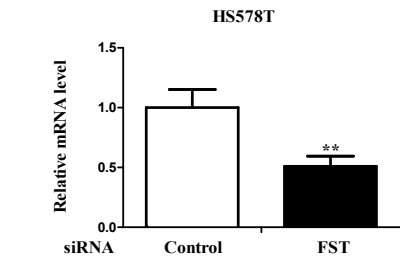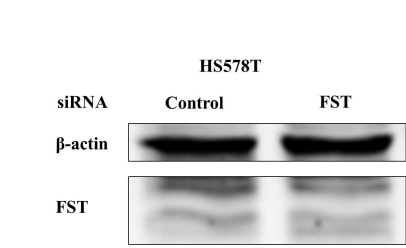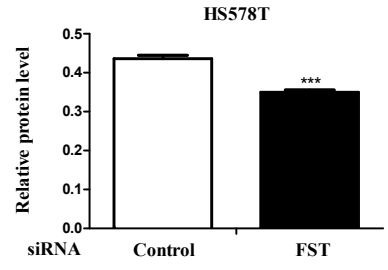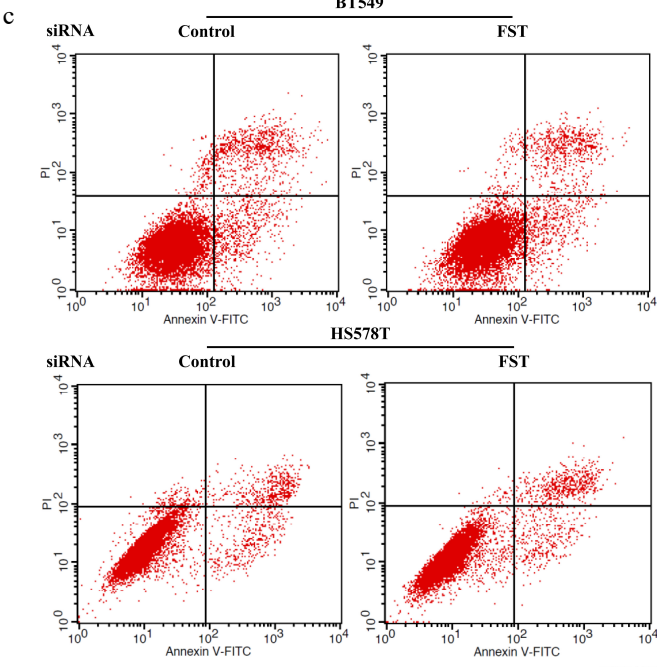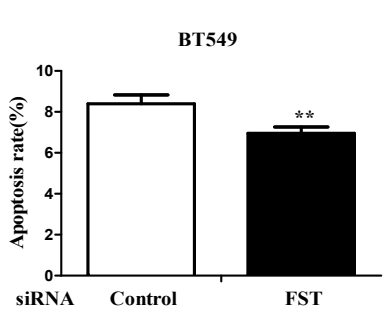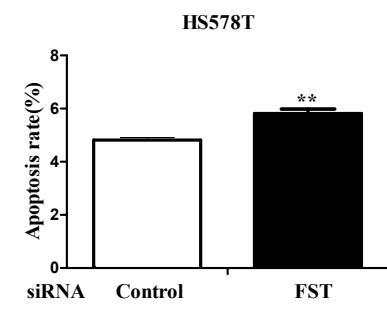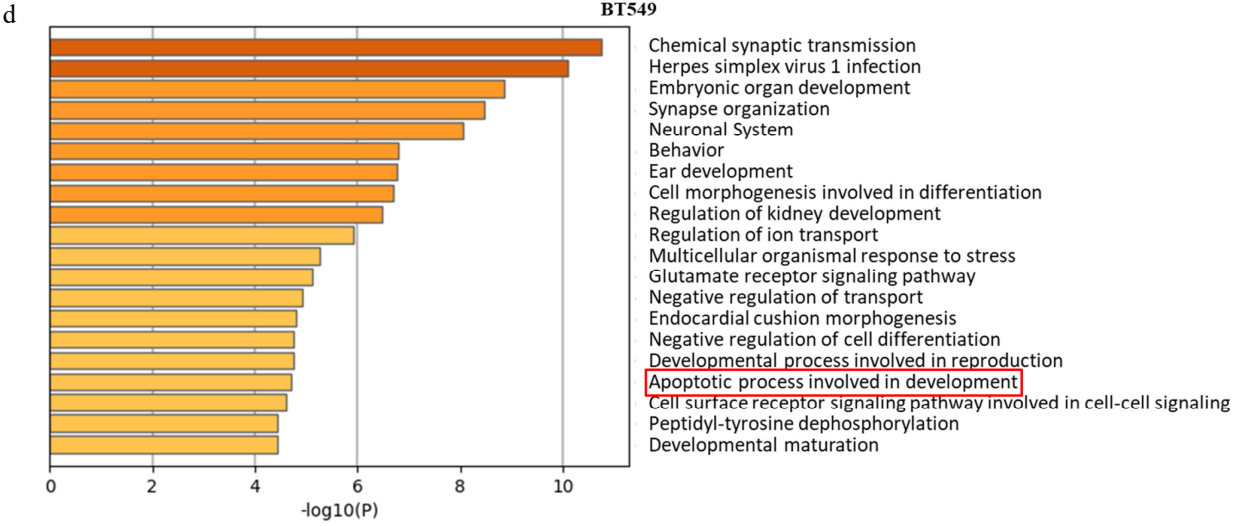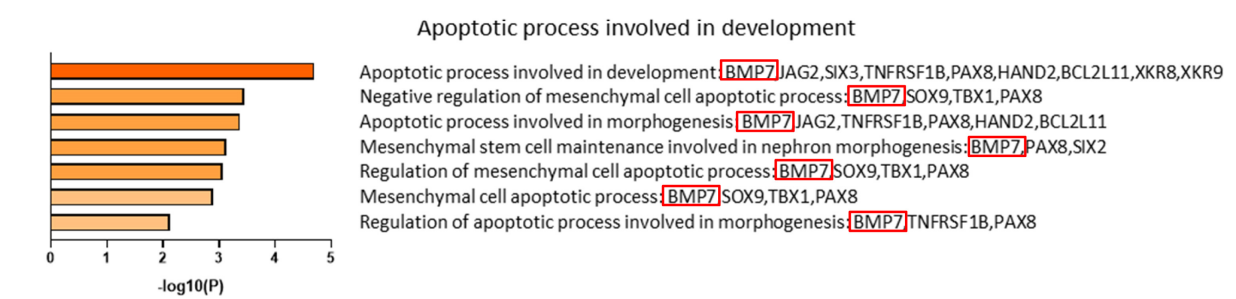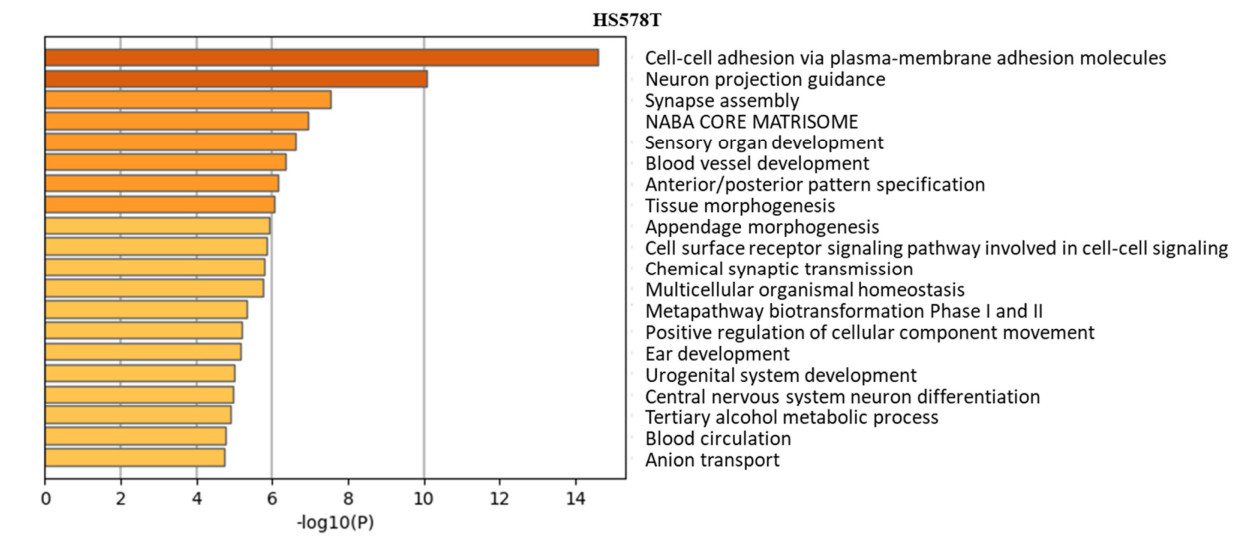

e

BT549

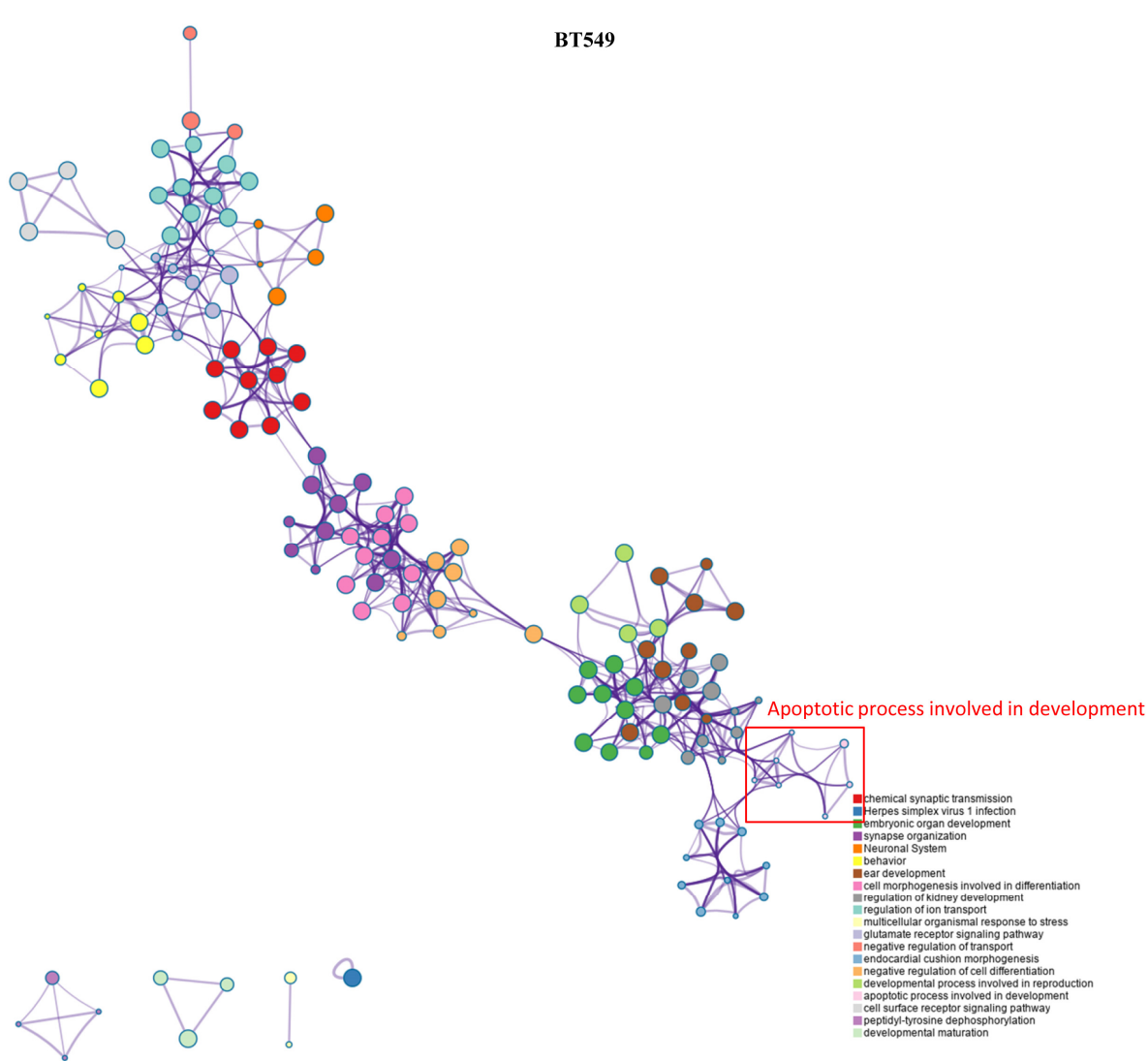

HS578T

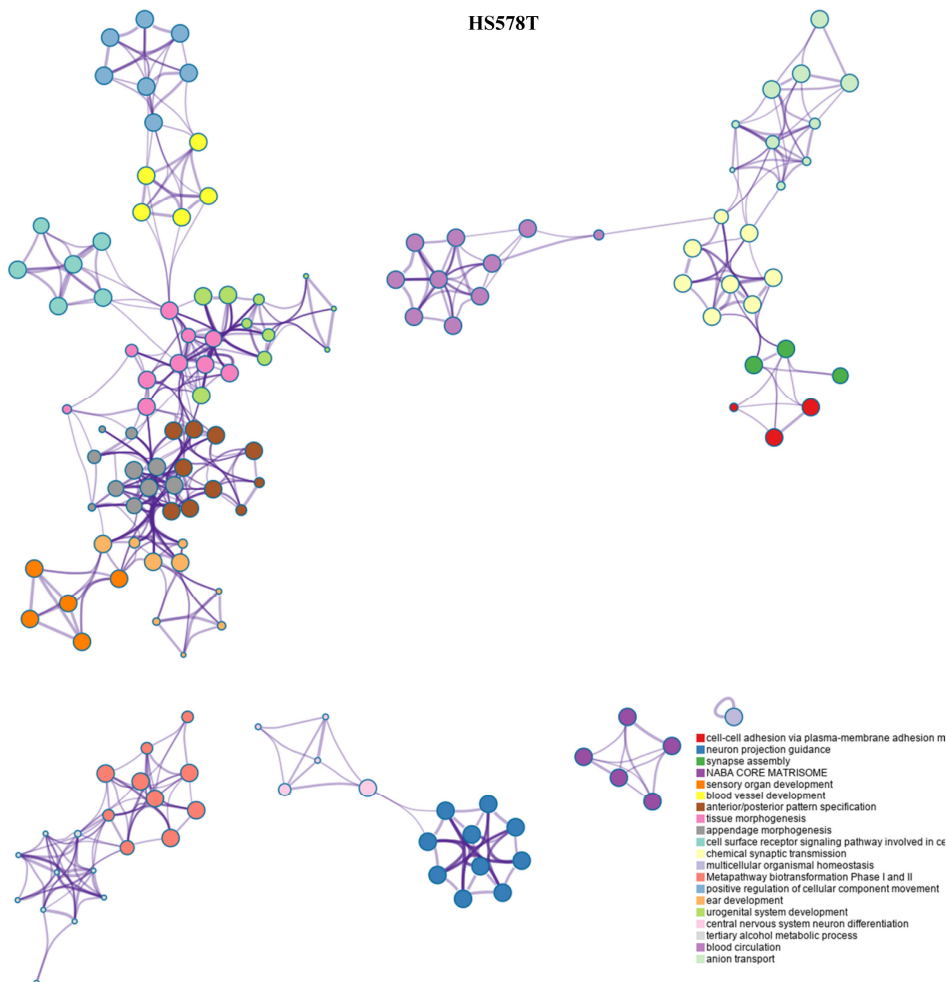

f

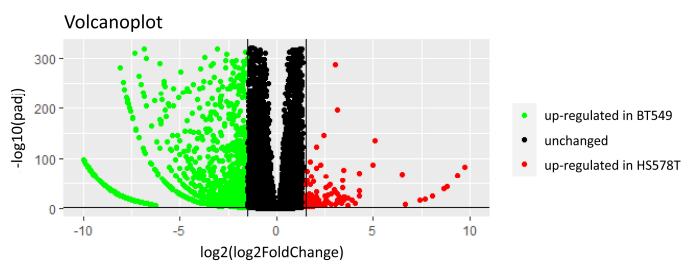

g

BMP7

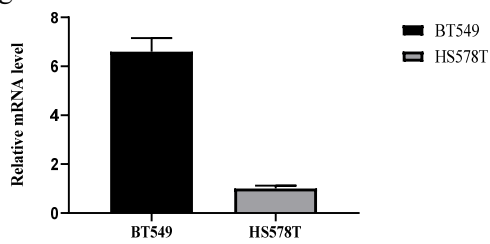

h

BT549

HS578T

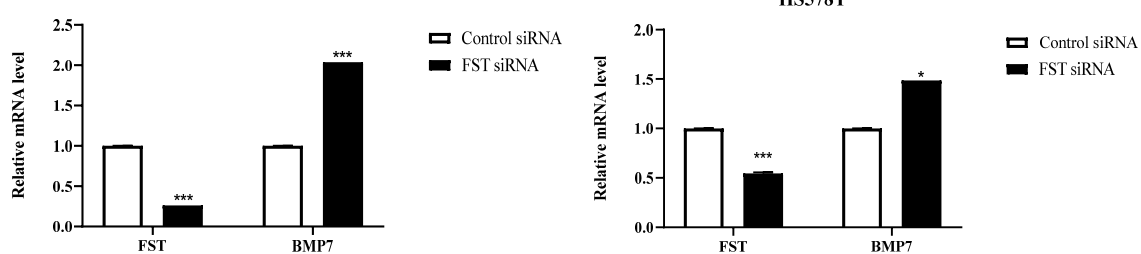

Supplement: Supplementary file 4 — Additional file 4: Figure S2. FST expression in siRNA targeted FST model and the role of FST in apoptotic pathways between BT549 and HS578T cells. [file 12935_2021_1977_MOESM4_ESM.pdf]
